# 

**Published:** 1883-08

**Authors:** 


CONTENTS.

| | Page. |
|---|---|
| Seasonable Clothing | 165 |
| Yellow Fever | 166 |
| "Constipation" of Nervous Origin | 169 |
| Spearing for Timber | 170 |
| The Christian Era | 171 |
| Stammering | 173 |
| Diphtheria and Nephritis | 174 |
| Swedish Women at Home | 175 |
| An Ancient Street | 176 |
| Opium Smoking | 176 |
| The Enduring Violin | 178 |
| Shooting Stars | 178 |
| Deafness from Tobacco Smoking | 179 |
| The Oldest Tree in the World | 179 |
| Rules in Case of Fire | 180 |

